# Microglial Lipid Biology in the Hypothalamic Regulation of Metabolic Homeostasis

**DOI:** 10.3389/fendo.2021.668396

**Published:** 2021-05-27

**Authors:** Andrew Folick, Suneil K. Koliwad, Martin Valdearcos

**Affiliations:** ^1^ Diabetes Center, University of California, San Francisco, San Francisco, CA, United States; ^2^ Department of Medicine, University of California, San Francisco, San Francisco, CA, United States

**Keywords:** microglia, hypothalamus, lipids, obesity, diabetes

## Abstract

In mammals, myeloid cells help maintain the homeostasis of peripheral metabolic tissues, and their immunologic dysregulation contributes to the progression of obesity and associated metabolic disease. There is accumulating evidence that innate immune cells also serve as functional regulators within the mediobasal hypothalamus (MBH), a critical brain region controlling both energy and glucose homeostasis. Specifically, microglia, the resident parenchymal myeloid cells of the CNS, play important roles in brain physiology and pathology. Recent studies have revealed an expanding array of microglial functions beyond their established roles as immune sentinels, including roles in brain development, circuit refinement, and synaptic organization. We showed that microglia modulate MBH function by transmitting information resulting from excess nutrient consumption. For instance, microglia can sense the excessive consumption of saturated fats and instruct neurons within the MBH accordingly, leading to responsive alterations in energy balance. Interestingly, the recent emergence of high-resolution single-cell techniques has enabled specific microglial populations and phenotypes to be profiled in unprecedented detail. Such techniques have highlighted specific subsets of microglia notable for their capacity to regulate the expression of lipid metabolic genes, including lipoprotein lipase (LPL), apolipoprotein E (APOE) and Triggering Receptor Expressed on Myeloid Cells 2 (TREM2). The discovery of this transcriptional signature highlights microglial lipid metabolism as a determinant of brain health and disease pathogenesis, with intriguing implications for the treatment of brain disorders and potentially metabolic disease. Here we review our current understanding of how changes in microglial lipid metabolism could influence the hypothalamic control of systemic metabolism.

## Introduction

The brain contains the second highest lipid concentration in the body, behind adipose tissue, and lipids constitute 50% of the brain’s dry weight (1). Beyond serving as energy substrates, brain lipids play a wide range of roles in cellular physiology, including membrane organization, protein modification, cell-cell interactions, membrane trafficking, energy storage and signal transduction. Lipid metabolism within the brain is therefore highly regulated, and disruption of central nervous system (CNS) lipid homeostasis can produce devastating neurological consequences. For instance, impaired cholesterol or fatty acid metabolism leads to severe neurodevelopmental defects, intellectual disabilities, and motor dysfunction (2, 3). Neurons themselves engage in relatively low levels of lipid synthesis, in contrast to recent studies which suggest that glial cells are critical for both the synthesis and metabolism of lipids in the brain (4). For example, an “astrocyte-neuron lactate shuttle” has been postulated, in which astrocytes metabolize lipids in order to provide energy substrates for neurons (5) and regulate neurite outgrowth and synaptogenesis (6). Oligodendrocytes are also highly active in lipid metabolism, and have been shown to synthesize the cholesterol necessary for myelin sheath formation (7). Importantly, microglia can both synthesize and accumulate lipids, and both microglial lipid composition and lipid metabolic capability are increasingly implicated in determining their ability to regulate neuronal functions as well as their contributions to brain pathology (8–10).

Integration of lipidomic and genomic datasets can elucidate gene-environment (e.g. diet) interactions regulating lipid metabolism as a means to reveal biomarkers predictive of metabolic disease (11). Recent studies utilizing lipidomics and single-cell RNA sequencing (scRNA-seq) have revealed intriguing heterogeneity among microglia, and the importance of lipid and lipoprotein metabolism in microglial physiology (12, 13). This work had been done primarily in the context of specific neurodegenerative diseases, while our lab and others have investigated the role of dietary lipids in the immunological activation of microglia in the context of obesity and metabolism (14–16). This review summarizes our current knowledge of lipid metabolism in microglia, with a focus on its potential contribution to hypothalamic physiology and dysfunction in the context of metabolic disease.

## Lipid Metabolism in the Brain

The brain has a high energy demand, and historical consensus has been that its energy requirements are almost entirely satisfied by glucose metabolism. However, this dogma has been recently challenged, as it was shown that approximately 20% of the brain’s total energy requirement is met though the oxidation of fatty acids (FAs) (17). Additionally, FA oxidation by cultured mouse brain slices is increased by withdrawing extracellular glucose (18). Astrocytes and microglia likely contribute significantly to brain utilization of FAs as energy substrates (18, 19). Astrocytes express higher levels of key FA oxidation enzymes, however detailed cell-type specific experiments comparing the capacity to oxidize fatty acids *in vivo* have not been reported (18). Neurons may have also the capacity to utilize FAs as an energy source, as a recent study using rat brain demonstrated that isolated neuronal mitochondria utilize FAs as an energy source even in the presence of other mitochondrial substrates (20). By contrast, the capacity of neurons to oxidize FAs for energy is known to be quite limited (21). One reason for this limited capacity may be that neurons are highly susceptible to reactive oxygen stress (ROS) generated by FA oxidation, and it is widely accepted that mitochondrial oxidative stress and dysfunction contribute to neurologic disorders (22). Thus, the selective pressure to avoid oxidative stress may underlie the neuronal preference to oxidize glucose as their primary fuel source (21).

### Neuron-Glia Interactions in Brain Lipid Metabolism

Given the importance of lipids to overall brain physiology, the limited lipid metabolic capacity of neurons themselves has prompted exploration into the essential roles of glial cells in lipid metabolism, storage and synthesis. This effort has revealed the importance of coordinated lipid metabolism and trafficking between neurons and glia, as exemplified by work done to establish a genetic link between Parkinson’s disease (PD) and genes controlling lipid metabolism (23, 24). Indeed, both PD patients and experimental animal models of PD exhibit abnormal lipid accumulation in dopaminergic neurons and their surrounding microglia, but have a reduced lipid load in adjacent astrocytes. One recent study found that a Western diet impairs recovery from demyelinating injuries, by inhibiting microglial phagocytosis and clearance of lipid debris (25). Another study found that in the setting of demyelination, microglia synthesize desmosterol, the immediate cholesterol precursor and liver X receptor (LXR) agonist, and that microglial sterol synthesis is essential for efficient remyelination (26). As oligodendrocytes were thought to be the primary synthesizers of sterols in the brain, and require sterols for myelination, this indicates a new role for intercellular trafficking of sterols. Together, these findings indicate that a disturbance in the multicellular handling and trafficking of lipids plays may play a key role in PD pathogenesis (27).

However a broader, more systematic understanding of the regulation of lipid metabolism and flux between brain cell types in different physiological and pathological states is limited, with few detailed lipidomic profiles of CNS cell types having been published to date. However, recent studies have revealed that certain lipid species are enriched in distinct cell types and brain regions. For instance, microglia are enriched in sphingolipids and characterized by high levels of sphingomyelin species, which are almost absent in neurons and oligodendrocytes (13). Microglia in particular are essential for the clearance and recycling of lipid debris, and recent work has shown that aging-related defects in microglial lipid handling contribute both to their inflammatory activation and to the impairment of their response to demyelination (28). Further insights into microglial lipid metabolism will be essential to understanding how lipids impact brain function.

### Effects of Diet on Brain Lipid Composition and Metabolism

In addition to having a relatively high absolute lipid content, the composition of brain lipids is also distinct from that of other tissues in the body. Indeed, 75% of lipids in mammals are present exclusively in neural tissues, underscoring that brain function has unique lipid requirements (29). The brain is the most cholesterol-rich organ in the body, and brain cholesterol is primarily supplied by local *de novo* synthesis (30). Cholesterol is essential for neuronal physiology, and defects in cholesterol metabolism leads to neurological diseases (31). Despite the primary *de novo* synthesis, diet may also affect sterol metabolism in the brain. The cholesterol metabolite 27-hydroxycholesterol (27-OHC) can pass through the BBB, and 27-OHC is significantly increased in plasma and adipose tissue of animals on HFD (32). Excess 27-OHC impairs brain glucose uptake (33). Additionally, peripheral cholesterol contained in circulating HDL, undergoes selective uptake mediated by the scavenger receptor class B type 1 for entry into the brain (34). In humans, low HDL levels are associated with increased risk for PD (35). Interestingly, genetic HDL deficiency caused increased astrogliosis, but not microgliosis, in the hypothalamus (36).

On the other hand, some FAs must be transported into the brain from the systemic circulation in a dynamic process (37). For instance, the brain is rich in long-chain polyunsaturated fatty acids (LC-PUFAs), particularly arachidonic acid (AA), eicosapentaenoic acid (EPA), and docosahexaenoic acid (DHA) but the brain has limited capacity to synthesize LC-PUFAs (38, 39). These have to therefore be provided through the diet, either as precursors, n-6 linoleic acid (LA) and n-3 α−linolenic acid (ALA), or as preformed AA and DHA (40, 41). Indeed, several radiolabeled studies have shown incorporation of circulating FAs into neurons (37). FAs could passively diffuse across the blood brain barrier (BBB), as shown for palmitate (PA), AA and DHA (42–44), however FA transporters such as FAT/CD36 may also play a key role in promoting the dissociation of FAs albumin in order to facilitate their diffusion across the BBB (45). In summary, brain lipid composition is highly regulated, and while distinct from peripheral lipid composition, is importantly influenced by circulating lipids including those from dietary sources.

The obesogenic high-fat diets (HFD) commonly used in mice, including the so-called “Western” diet with increased cholesterol levels, are characterized by a markedly high saturated fatty acid (SFA) content and a relatively low n-3 polyunsaturated fatty acids (PUFA) content, resulting in a high n-6/n-3 ratio (46). Given this, it is notable that studies suggest that not every type of fat is equally obesogenic when consumed in an isocaloric manner. Indeed, the profile of consumed fats, rather than strictly the energy they contain, may be critical for the development of obesity (47). Circulating lipid levels are affected by dietary fat composition; for example, one lipidomic analysis of postprandial plasma showed significant changes in the levels of 316 different lipids species after an individual switched from eating a breakfast based on dairy foods to one that was soy oil-based (48). Recent studies have also investigated the effect of dietary fat on the brain lipidome. Mice consuming a HFD have reduced EPA content in cerebral phospholipids and sphingolipids, in association with increased inflammation and consequently impaired brain function (49). By contrast, diets enriched in the n-3 fatty acids EPA and DHA induce a different set of alterations in the FA composition of brain phospholipids, including increasing the number of double bonds in several phospholipid species (13). Indeed, supplementing a standard saturated fat-rich HFD with a daily gavage of fish oil rich in EPA and DHA is sufficient to increase brain PUFAs and reduce brain gliosis in obese mice (50). In probing this further, it is notable that both EPA and DHA are precursors of pro-resolving lipid mediators with anti-inflammatory properties (51). In contrast, n-3 deficient neonatal mice exhibit increased microglial phagocytosis of synaptic elements resulting in altered neuronal morphology and function (10). Thus, dietary changes in EPA and DHA, as detected by monitoring dietary n-6/n-3 ratios, may directly modulate microglial polarization states in a manner relevant to CNS diseases associated with microglial dysfunction.

## Integration of Lipid Signals by the Mediobasal Hypothalamus (MBH)

The MBH, defined here as the hypothalamic region containing the arcuate nucleus (ARC) and median eminence (ME), is strategically located to directly sense and coordinate a response to nutritional signals. The structure and function of the BBB within the ME and ventromedial ARC, as a circumventricular organs, is unique, being supplied by fenestrated capillaries (52–55). Thus, substances that do not cross into the brain parenchyma in other regions of the brain may pass into the ME and ARC with relative ease (56, 57). For example, very low density lipoproteins (VLDL) are not thought to cross the BBB (34), however we demonstrated rapid accumulation of VLDL within the MBH after intravenous administration, and this was predominantly localized to microglia in the ME and ARC (14). Indeed, a recent study showed that triglyceride (TG)-rich lipoproteins are sensed in the hypothalamus by an LPL-dependent mechanism (58), and the uptake of dietary PA, a common SFA, into the hypothalamus is remarkably higher than for other brain regions (59). Thus, specialized fuel-sensing neurons that form critical hypothalamic circuits are uniquely positioned to sense circulating glucose and lipid species, including FAs (60).

Whereas lipid sensing in the MBH may create responsiveness to nutritional lipids, there may be roles for lipid sensing in other brain regions as well. For instance, recent work has shown that both nutritional and parenteral TG exposure modulated activity of neurons in the mesocorticolimbic system (MCL) and affects behavioral and reward responses (61, 62). These effects were dependent on neuronal lipoprotein lipase, suggesting a direct response to TGs (61, 62). Radiolabeled triolein was able to be detected in whole brain after peripheral injection, suggesting that some intact TG may pass through the BBB, however the location of triolein accumulation within the brain was not determined (63). This sensing capacity may play a role in the context of autophagy, lipids housed within locally-generated lipoproteins (e.g. APOE), or perhaps by context-specific selective permeability of the vasculature in certain brain regions to circulating lipids. Further research is needed to tease apart these possibilities.

### Lipid Sensing by Hypothalamic Neurons

Circulating FA levels are increased after consumption of a HFD (64, 65) and the rate of entry of FAs into the brain is proportional to their plasma concentration (59). Indeed, hypothalamic levels of free FAs are increased by HFD feeding, suggesting an important role for these FAs in hypothalamic lipid-sensing pathways (66). Supporting this, it has been shown that FAs modify neuronal firing rates in the ARC (67). Moreover, intracerebroventricular (ICV) infusion of the monounsaturated FA, oleic acid (OA), suppresses food intake and hepatic glucose production (68) indicating that FAs can signal nutrient availability to the brain. Furthermore, increased LCFA-CoA levels in hypothalamic neurons suppress endogenous glucose production suggesting that hypothalamic lipid sensing regulates glucose homeostasis through a mechanism involving the esterification of LCFAs to LCFA-CoAs (69). Also, ICV infusions of OA or DHA, but not PA, reduce food intake and body weight indicating a selective hypothalamic response to specific unsaturated fatty acids (UFAs). However, ICV and direct infusions of FAs into the brain are not physiological. Short-term (3 days) of HFD can cause rewiring of anorexigenic proopiomelanocortin (POMC) neurons in the ARC, suggesting a physiological role for lipid sensing in the hypothalamus (70). In further support of this concept, a recent elegant study found that intragastric administration of lipids inhibited the activity of hunger-promoting Agouti-related protein (AgRP) neurons in the MBH (71). Furthermore, the ability of lipid infusion to inhibit the activity of AgRP neurons was blunted in HFD-fed animals, suggesting a reduction in the lipid sensitivity of AgRP neurons in this context (72). In summary, there is clear evidence that a HFD, in particular dietary FAs, is sensed by hypothalamic neuronal pathways to regulate energy homeostasis. The precise mechanisms of lipid sensing by hypothalamic neurons have been well studied, and have been recently reviewed (73–75).

Dysregulated lipid metabolism in the hypothalamus may affect neuronal FA sensing and therefore contribute to the development of metabolic diseases. In particular, excessive lipid accumulation and resultant activation of cellular stress pathways can lead to disruption of hypothalamic function. During both acute and chronic HFD feeding, multiple inflammatory and stress response pathways are activated in the hypothalamus, leading the dysfunction of hypothalamic circuits regulating energy and glucose homeostasis, resulting in leptin and insulin resistance (76). In evaluating the specific changes in hypothalamic lipid composition induced by overconsumption, specific attention has been paid to how excess lipid accumulation drives ER stress in the hypothalamus (77). Rodent studies have shown that HFD feeding induces ER stress in multiple metabolic tissues including the hypothalamus (78). This response is not uniform across all hypothalamic nuclei and seems to be specific to the ARC but not other regions such as the paraventricular nucleus (PVN) (78). Induction of hypothalamic ER stress leads to increased food intake, reduced energy expenditure and resultant obesity, and this is mediated at least in part by defective α-MSH production among POMC neurons (79) and development of leptin resistance (80). Fat composition is important in this regard, because saturated fats (e.g., PA) are more deleterious than unsaturated fats to hypothalamic neurons (81), and ER stress sensors are specifically activated by increasing ER membrane lipid saturation (82). Interestingly, PA-induced ER stress in hypothalamic neurons decreases protein abundance and function of the melanocortin 4 receptor (MC4R) (83), and central inhibition of lipid oxidation and ER stress is sufficient to restore hypothalamic lipid sensing and energy homeostasis in mice (84).

ER stress and inflammatory pathways are functionally coupled, and induction of CNS ER stress in lean mice is sufficient to activate NF-κB signaling (85). Furthermore, there is convincing evidence that ER stress activates the NLRP3 inflammasome in myeloid cells through different pathways in a context-dependent manner. We demonstrated that IRE1α, a critical ER sensor of both unfolded protein and saturated lipid stress, mediates SFA-induced IL-1β secretion in macrophages upon sensing increasing saturation of cellular phospholipids (86). However, most metabolic studies in the hypothalamus have focused on neuronal ER stress, and the potential contribution of ER stress in glial cells to hypothalamic dysfunction has not been explored yet. However, a recent study did show that disrupting proteasome activity in microglia triggers the induction of a type I interferon (IFN) response in an IRE1-dependent manner (87), suggesting that microglial ER stress is worth studying in the context of hypothalamic regulation.

### Lipid Sensing by Non-Neuronal Cells

Hypothalamic neurons are critical to the regulation of energy and glucose homeostasis, and our understanding of neuronal circuits controlling metabolism has advanced greatly over the past decade. However, recent studies implicate non-neuronal cells, including microglia, as physiologic regulators of hypothalamic function as well. For instance, astrocytes are the most abundant glial cells in the CNS and are involved in multiple fundamental processes, including metabolic homeostasis, neurovascular coupling, and BBB maintenance (88). Recent studies show that disrupting astrocyte lipid homeostasis may contribute to neurological disorders (89, 90). In addition, astrocytes participate in immune responses, and HFD consumption induces morphological changes in hypothalamic astrocytes (91). Astrocytes can influence hypothalamic circuits involved in the control of feeding and energy metabolism, at least in part by regulating extracellular levels of adenosine (92, 93). Furthermore, a recent study suggested that astrocytic insulin signaling regulates hypothalamic glucose sensing and systemic glucose metabolism (94). Also, astrocytes in the MBH can respond to acute changes in nutritional exposure, with morphological changes after overnight fasting (91), or as soon as 1-hr post-prandially (95). Interestingly, post-prandial retraction of astrocytes surrounding POMC neurons was only seen with standard chow diet, but not HFD (95). Tanycytes are radial glia-like cells that line the wall of the third ventricle in the brain, a privileged position to integrate multiple peripheral inputs (96, 97). Tanycytes can sense nutrients such as FAs in the cerebrospinal fluid (CSF), facilitate the transport of metabolic hormones across the BBB, and integrate signals to regulate appetite and energy balance (98, 99).

Microglia are increasingly being recognized as highly dynamic cells that continuously monitor for alterations to their environment, and assume different states of activation according to the unique CNS microenvironment in which they reside. Within the hypothalamus, microglia are emerging as key physiological mediators, both in the context of normal hypothalamic function and regulating the metabolic response to HFD. As such, key details of microglial lipid sensing and metabolic regulation have gained considerable interest, and are therefore reviewed below.

## Microglia as Novel Regulators of Hypothalamic Function

**Figure 1:**
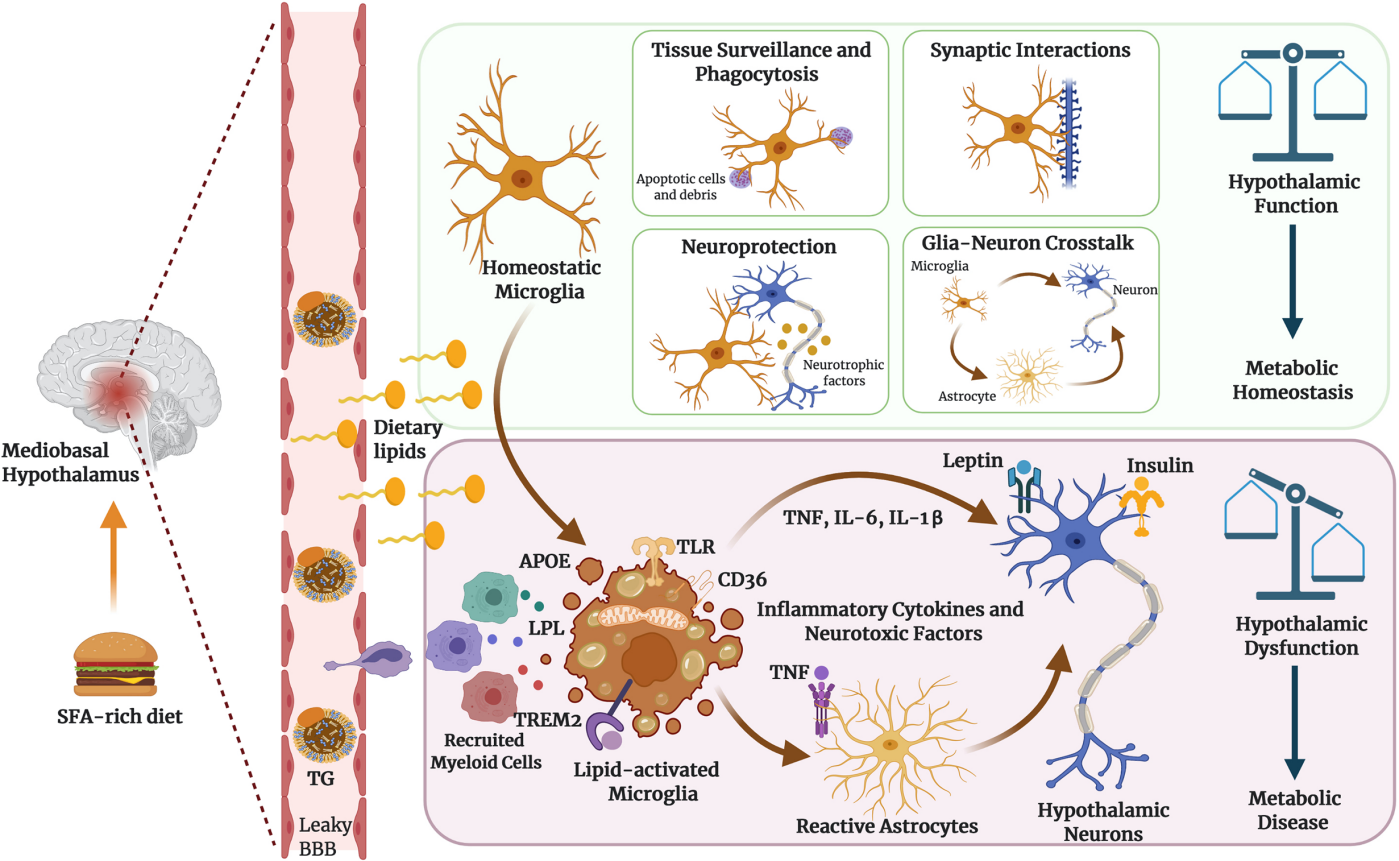
Microglia as dynamic cellular mediators of hypothalamic function. Microglia can perform diverse functions to maintain brain homeostasis, actively screening the surroundings, intercellular communication and remodeling the brain circuits through synaptic pruning and neuronal plasticity. Hypothalamic microglia integrate systemic metabolic signals such as dietary lipids to establish functional states that influence neuronal control of energy homeostasis. TG: refers to triglyceride-rich lipoproteins, including chylomicrons and VLDL particles. Figure created with BioRender.com.

Recent findings reveal an expanding array of functions for microglia, beyond their established roles as immune sentinels and phagocytic removers of cellular debris. These include roles in synaptic organization (100), neuronal excitability (101) and trophic support for brain repair (102) *(* **Figure 1**). Given the vital role of microglia in maintaining CNS homeostasis, it is not surprising that several brain disorders are associated with microglial dysfunction (103). Disrupting the interactions between neurons and microglia has devastating effects on memory, anxiety and other behavioral domains, demonstrating the importance of myeloid cells in brain physiology (104). Furthermore, interactions between microglia and astrocytes have been implicated in brain health and disease (105), and their cross-talk may play an important role in HFD-induced hypothalamic dysfunction. Activated microglia can induce reactive astrocytes by secreting proinflammatory molecules, such as IL-1α, TNF and C1q as previously demonstrated in a lipopolysaccharide (LPS)-induced murine neuroinflammation model (106). In addition, recent work suggests that microglial activity is directly regulated by metabolites of dietary tryptophan metabolism produced by commensal flora, and that this response controls a downstream inflammatory response among astrocytes (107). Recently, we showed that the inflammatory signaling of microglia dictates susceptibility to diet-induced hypothalamic dysfunction and obesity (15).

### Microglial Inflammatory Signaling Regulates Hypothalamic Immune Response to Dietary Excess

Chronic low-grade inflammation is considered one of the hallmarks of metabolic disease, and activation of inflammatory pathways have been described in several metabolic tissues. Animal and human studies have identified white adipose tissue (WAT) as the primary site where inflammation is initiated and exacerbated in response to weight gain (108). Obesity promotes drastic changes in the resident immune cell profile and function in WAT. Adipose tissue macrophages adopt a metabolically activated (MMe) phenotype distinct from that associated with classical “M1” activation, upregulating proteins involved in lipid processing including ABCA1, PLIN2 and CD36 to maintain adipose tissue homeostasis (109). Moreover, a novel and conserved macrophage population called lipid-associated macrophages (LAMs) is involved in controlling WAT lipid homeostasis has been recently described in multiple obesity-related mouse models (110).

Interestingly, recent studies have provided evidence that HFD consumption also increases the expression of genes governing inflammatory signaling in the hypothalamus (111–113). This phenomenon has also been described in human obesity, and obese individuals without a systemic disease showed markedly increased levels of inflammatory markers in the hypothalamus compared to healthy non-obese individuals (114, 115). With this in mind, it is notable that HFD consumption in mice rapidly increases the accumulation and activation of microglial populations secreting inflammatory cytokines specifically in the MBH (14). Moreover, the activation of hypothalamic inflammatory pathways in response to HFD consumption is much more rapid than it is in peripheral tissues such as WAT, even preceding any significant diet-induced weight gain, suggesting that the inflammatory response of the MBH to dietary excess is a cause, rather than a consequence, of obesity (113). Indeed, a single high-fat meal is sufficient to induce morphological changes and increased *Iba1* expression in hypothalamic microglia (16).

We have shown that either pharmacologically depleting resident microglia, or genetically restraining their inflammatory capacity *via* NF-κB signaling, protects mice from diet-induced hyperphagia and weight gain, whereas specifically forcing NF-κB-dependent microglial inflammatory activation reduces energy expenditure and increases both food intake and weight gain even in absence of a dietary challenge (15). Microglial inflammatory signaling may induce obesity by causing hypothalamic neuronal dysfunction, including the induction of neuronal insulin and leptin resistance (76). Moreover, prolonged microglial activation may also induce apoptosis of anorexigenic/catabolic POMC neurons (116).

### Metabolic Plasticity of Microglia

**Figure 2:**
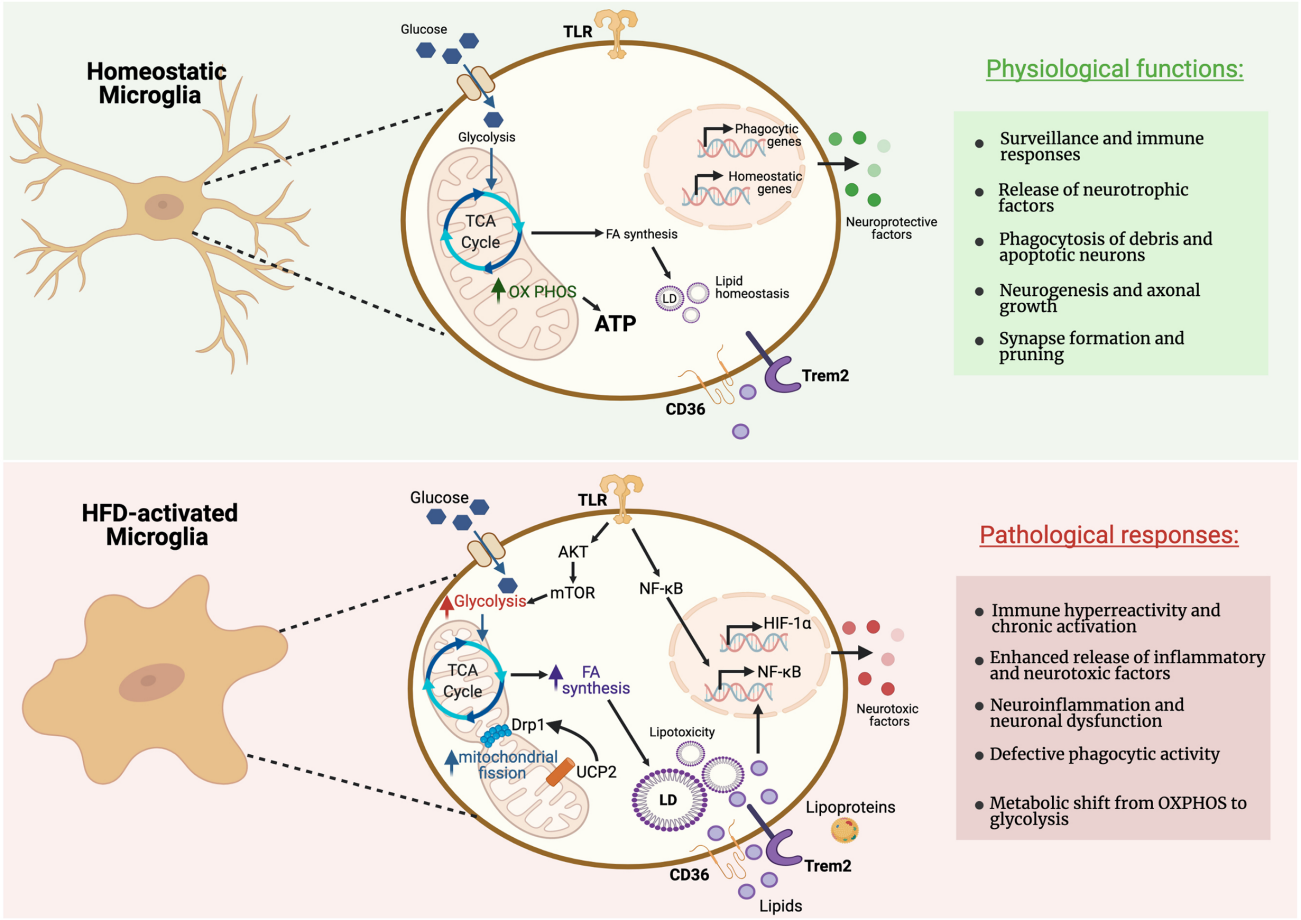
Metabolic pathways regulating microglial activity during homeostasis and pathological responses. Microglia can rapidly adapt their energy metabolism to nutrient availability and transcriptomic analyses revealed that microglia express genes necessary for both glycolysis and oxidative metabolism. Microglia in their homeostatic status show reliance on oxidative metabolism to maintain their neuroprotective properties. However, microglia in proinflammatory states preferentially use glycolysis for energy production. This metabolic switch towards glycolysis allows microglia to produce ATP rapidly, despite being comparatively less efficient, for the secretion of inflammatory cytokines. High-fat diet (HFD) triggers a microglial inflammatory response leading to neuronal dysfunction in the MBH. Figure created with BioRender.com.

Microglia have the ability to adapt their metabolic pathways to use the energy substrates available in their local environment, and to acquire diverse and complex phenotypes during inflammatory activation in response to an insult or injury (18)(**Figure 2**). A comparative transcriptional profiling of genes related to energy metabolism in different brain cell types revealed that microglia express specific sets of genes required for both glycolytic and oxidative energy metabolism (117). For instance, microglia express the long-chain fatty acyl-CoA synthetase, which catalyzes the formation of fatty acyl-CoAs that are, in turn, β-oxidized into acetyl-CoA units and can be further metabolized in the TCA cycle. Additionally, a recent study showed that microglia are able to maintain oxidative phosphorylation and homeostatic function during periods of hypoglycemia by shifting fuel utilization to glutamine (18). Homeostatic microglia, which are tasked with regulating day-to-day aspects of tissue homeostasis throughout the CNS, rely mainly on oxidative phosphorylation for ATP production, while microglia activated in the context of pro-inflammatory circumstances favor glycolysis (118, 119). When specifically activated, microglia are able to release several metabolites into the extracellular milieu (e.g.: succinate, itaconate, lactate) that modulate neuronal functionality and survival. For instance, a recent study showed that succinate produced by CNS myeloid cells is sensed by neural stem cells during the chronic phase of a mouse model of experimental autoimmune encephalitis (EAE) to ameliorate neuroinflammation *via* succinate-dependent mechanisms (120). Experiments on cultured microglia consistently show that they respond to proinflammatory stimuli by increasing glycolytic flux (121, 122). The metabolic alterations of isolated cells *in vitro* may differ from those *in vivo*. However, a novel approach to image NADH fluorescence has been recently employed to detect an enhanced glycolytic response of microglia to LPS treatment in mouse brain slices (18). Glycolysis is less efficient than oxidative phosphorylation (OXPHOS), however this glycolytic shift may redirect metabolites to provide the cell with precursor molecules for the production of inflammatory factors. Indeed, it has been shown that glycolysis is indispensable to stimulate secretion of pro-inflammatory cytokines by macrophages, the peripheral tissue analogs of microglia (123). Conversely, fatty acid β-oxidation and mitochondrial function are necessary for microglia to manifest relatively anti-inflammatory polarization states (124).

In considering what might control broad shifts in fuel metabolism among microglia, it is notable that epigenetic changes, including histone modifications and DNA methylation, are important modifiers of gene expression and are known to mediate the metabolic reprogramming of myeloid cells. For instance, feeding mice a HFD for 4 weeks is sufficient to induce lasting epigenetic modifications in myeloid progenitor cells in the bone marrow, leading to increased immune responses to LPS challenge even after the mice were returned to a regular low-fat chow diet (125). Despite the fact that metabolic alterations have been implicated in several disease models (126), more knowledge is needed to understand which specific metabolic pathways can be targeted to restore the homeostatic microglia phenotype in chronic inflammatory diseases, including obesity.

Recent studies have shed light on how changes in mitochondrial morphology and function may impact microglia polarization and function. Microglia stimulated with LPS, demonstrate increased mitochondrial fragmentation, which was dependent on ROS-mediated activation of adenosine monosphosphate-activated protein kinase (AMPK) (127). Mitochondrial fragmentation in reactive microglia requires dynamin-related protein 1 (DRP1), an essential component of mitochondrial fission (127). Short-term HFD (3 days) caused decreased size and increased number of mitochondria in microglia in the MBH, associated with increased levels of activation of DRP1 (128) (**Figure 2**).

Mitochondrial uncoupling protein 2 (UCP2) plays a key role in reactive microglia. Knockdown of UCP2 modulates microglia response to both LPS and IL-4 (129). Deletion of microglial UCP2 prevented HFD-induced increases in mitochondrial fission in MBH microglia, and reduced microglial activation in the MBH and HFD-induced obesity (128). UCP2 has been shown to effect both ROS production (130, 131) and fuel utilization (132). Microglia in culture showed increased mitochondrial respiration in the presence of high glucose and palmitate, dependent on the presence of UCP2 (128). Further mechanistic studies are necessary to explore the impact of mitochondrial function and fuel utilization in the regulation of MBH microglia.

### The Impact of Sex on Microglial Phenotypes in Metabolic Regulation

The study of sex differences in physiology has gained attention, and sexual dimorphism in obesity and metabolic disease has been described (133, 134). HFD induces activation of microglia in the MBH of rodents, in a sexually dimorphic manner, affecting male differently than females (135). However, the mechanism underlying these differences are not well understood. Interestingly, recent studies suggest that male and female mice differentially metabolize lipids acquired from the diet. For instance, HFD feeding increases PA and sphingolipids levels in the hypothalamic tissue of male mice but not in the females (135). Alterations in sphingolipid-mediated signaling pathways might provide an additional mechanism by which SFAs induce hypothalamic dysfunction in the MBH (136). On the other hand, microglia in the adult mouse brain have sex-specific features and that could explain sex differences in neurological disease susceptibility (137). Moreover, it has been recently shown that microbiota influences adult microglia in a sex-specific manner (138). For instance, short-chain fatty acids (SCFAs) are the main metabolites produced by bacterial fermentation of dietary fiber in the gastrointestinal tract, and these SCFAs influence gut-brain communication and brain function directly or indirectly through immune, endocrine, and vagal pathways (139). Although the SCFAs have been shown to protect against diet-induced obesity in mice (140) and overweight humans (141), the underlying mechanisms are not well understood. SCFAs are important regulators of innate immune responses and recently have been involved in the regulation of microglial function (142). Thus, regulating CNS myeloid cell functions by manipulating the gut microbiota may represent a promising therapeutic approach to mitigate metabolic diseases.

## Dietary Lipids Regulate Microglial Polarization and Responses in the MBH

Bioactive dietary FAs are potent modulators of microglial inflammatory responses. Lipid accumulation in myeloid cell types more broadly, is well demonstrated to be associated with the activation of inflammatory signaling cascades (143). Moreover, microglia express a wide range of lipid metabolism-related genes such as those encoding fatty acid oxidation enzymes (144), lipoprotein lipases, lipid transporters, and lipid-sensitive receptors (e.g. receptors for endocannabinoids, prostaglandins or phospholipids), suggesting that lipids are important regulators of microglial physiology. Microglia can store FAs within lipid droplets, which are known to control their inflammatory responsiveness and phagocytic activity (145). Some reports suggest that dietary lipids in the context of the whole mammal, can also influence microglial function through indirect mechanisms including microbially-derived metabolites, hormonal control, and gut and systemically-derived inflammatory signals. For instance, treating microglia with insulin *in vitro* decreases LPS-induced TNF production and phagocytic activity in a dose-dependent manner (146). Moreover, ghrelin, an orexigenic hormone produced by the stomach and duodenum, directly exerts anti-inflammatory and anti-oxidative effects on LPS-activated microglia when introduced to them in culture (147).

In particular, long-chain SFAs have emerged as a potential nutritional triggers of microglial activation in the MBH, exerting effects in the brain analogous to those documented for peripheral tissues. HFD intake increases brain SFA levels, and more specifically those of lipids containing PA (14). Indeed, PA levels are increased in the CSF of overweight and obese humans (148). We showed that microglia in the MBH can sense rising levels of saturated fats, when consumed in excess, and transduce this to instruct local neurons. Moreover, enteric isocaloric gavage of SFAs, but not UFAs, for only 3 days is sufficient to induce microglial activation in the MBH, reproducing the response seen in the MBH of mice fed a HFD (14). These findings support the idea that SFAs trigger this response. However, the HFD commonly used for animal studies also contains high amounts of sugars, and another study suggested that dietary sugars, instead of fat, drive hypothalamic inflammation (149). One caveat of this study was that the authors did not control for calories and the sources of fat *vs*. carbohydrates across diets. Intriguingly, a recent comprehensive study of 29 different diets with different macronutrient compositions showed that only dietary fat, but not protein or carbohydrates, regulates hypothalamic control of energy intake and promotes adiposity (150). Besides macronutrient distribution, the specific source of dietary FA is can modulate microglial inflammatory responses. For instance, the substitution of dietary lard for flaxseed oil or olive oil reduced food intake and inflammatory markers in the MBH, highlighting a specific pro-inflammatory impact of SFAs (151).

SFAs were initially thought to induce inflammation as direct agonists of the toll-like receptor 4(TLR4), a member of the interleukin-1 receptor superfamily with a prominent role in innate immune responses. In support of this hypothesis, pharmacological and genetic approaches to inhibit hypothalamic TLR4 signaling suppressed SFA-induced microglial activation and inflammatory cytokines expression in rodent models fed a HFD (112, 152). However, a recent study showed that TLR4 is not the receptor for SFAs. Rather, TLR4-dependent priming alters cellular metabolism, lipid metabolic pathways and membrane lipid composition, changes that are required for engagement of SFA-induced inflammatory pathways (153). The fatty acid translocase CD36 is another potential mediator of microglial lipid-sensing. Indeed, CD36 has been shown to be essential for microglia-mediated uptake of myelin debris (154), and microglia response to beta-amyloid (155). However, while CD36 is known to be involved in long-chain fatty acid uptake and sensing in other tissues, its role in lipid-sensing in microglia has not been reported. Unlike SFAs, PUFAs have beneficial effects on the brain and reduce neuroinflammation. PUFAs, when incorporated into cell membranes, increasing membrane fluidity in a manner that was shown to help microglia engage in phagocytosis (156). Microglial movement was remarkably impaired in mice fed a diet deficient in n-3 PUFAs (157). Also, PUFAs are endogenous ligands of the G-couple receptor GPR120, which may explain, at least in part, how they activate anti-inflammatory signaling pathways (158). Moreover, GPR120 is primarily expressed by microglia in the hypothalamus and is suggested to be involved in regulating microglial inflammatory responses that influence energy homeostasis (159).

## Lipoprotein Metabolism and Lipid Mediators Regulating Microglial Phenotypes

The emergence of new technology such as scRNA-seq has enabled the identification and characterization of the diversity of microglial populations. These studies have revealed that the heterogeneity of microglia in both normal and disease states exists beyond the simplistic M1/M2 paradigm, with a spectrum of cellular states existing from homeostatic microglia to pathology-associated microglia (160, 161). In addition, scRNA-seq of myeloid cells has revealed extensive regional heterogeneity in both microglia and non-parenchymal brain myeloid cells including so-called “border-associated” macrophages found proximal to, and within, meningeal lining tissue (162). Recently, several comprehensive *ex vivo* scRNA-seq analyses of microglia have defined specific transcriptional clusters with common metabolic characteristics. For instance, a novel microglial population called disease-associated microglia (DAM) was recently identify in a mouse models of AD and amyotrophic lateral sclerosis (ALS) expressing a distinct set of genes associated with lipid and lipoprotein metabolism (163). This transcriptional signature represents a preference for lipids as fuel substrates, ostensibly to meet the increased bioenergetic demands of this form of activated microglia (163). A similar signature is also observed in microglia in the context of demyelination, suggesting engagement of a transcriptional microglial phenotype that enables the ability to phagocytose and clear lipid debris (164). Moreover, human microglia from white matter adjacent to chronic multiple sclerosis (MS) lesions showed upregulation of scavenger receptor and lipid metabolism genes including *LPL* and *PPARG (* 165). Additionally, analyses of non-diseased human brain revealed clusters of microglia enriched for expression of metabolism-encoding genes, including *APOE* and *LPL*, in white- *vs*. grey matter, and increased with aging (166). While the transcriptional signature of activated microglial populations in these studies have shown variability in the response to different stimuli and experimental conditions, there is a clear consistent implication of alterations in lipid metabolism in analyses of microglia activated by stimuli other than those associated with acute infection.

### Lipoprotein Lipase (LPL)

Lipoprotein lipase (LPL), an enzyme needed for the hydrolytic cleavage and release of FAs from TGs, and a number of recent reports have highlighted LPL as a key feature of reparative microglia, which are recruited to restore tissue homeostasis in the context of injury, for example. scRNA-seq of DAM, in a murine model of Alzheimer’s disease (AD), revealed that *LPL* levels are markedly increased in a unique microglial subset associated with phagocytosis and protection in AD (163). Furthermore, *LPL* gene transcription is elevated in a cuprizone model of demyelination (167), and a recent study suggested that LPL is a novel feature of a the supportive microglial phenotype that emerges during remyelination and repair *via* clearance of lipid debris (9).

LPL is expressed in the brain, spinal cord, and peripheral nerves but is predominantly expressed by macrophages and microglia in the human and murine brain (117, 168). Although the function of LPL in the microglial response to neurodegenerative disease is not well understood, *LPL* polymorphisms are been implicated in disease risk, such as an association with AD risk. For instance, loss-of-function *LPL* polymorphisms with reduced enzymatic activity are associated with increased AD risk as well as with increased VLDL-TG levels (169). Conversely, patients with *LPL* polymorphisms leading to increased LPL activity have reduced hippocampal amyloid plaque formation (170). Microglia-specific knockdown of *Lpl* exhibited decreased cell number and soma size of microglia in the ARC of mice fed a hypercaloric diet (168), supporting the hypothesis that lipoprotein metabolism is important in the regulation of MBH microglial function. In these mice, POMC neuronal loss was accelerated and they gained more weight than control mice. Microglia lacking *Lpl* demonstrated a shift in fuel utilization towards glutamine and decreased phagocytic capacity, suggestive of an immunometabolic shift (168). Taken together, these data suggest that LPL regulates lipid and lipoprotein uptake, which may provide the lipids needed to maintain homeostatic microglial functions in the MBH.

### Apolipoprotein E (APOE)

Apolipoprotein E (APOE) is the major carrier for lipids in the brain, and *APOE* genotype is the most profound genetic risk factor for AD, predominantly by modulating microglial activation (171). In the brain, APOE is expressed predominantly by astrocytes and microglia and a major role for APOE in the brain is to maintain a consistent supply of essential lipids to neurons (172). Extensive studies have established the role of APOE in mediating inter-cellular cholesterol transport from glia to neuronal cells (173). The human APOE gene exists as three different alleles, ε2, ε3 and ε4 and these isoforms change the lipid and receptor binding ability of APOE.

Microglial APOE production is strongly induced during injury and disease, including in AD (174). APOE is a key component of transcriptional signature of activated microglia, as demonstrated in post-mortem human brain studies, AD mouse models and studies of cultured microglia (163, 171). APOE induces an anti-inflammatory phenotype in macrophages and similarly an APOE peptide inhibits inflammatory processes in isolated microglia through the APOE receptor, LRP1 (175). In APOE-deficient mouse models, peptides based on the APOE receptor-binding domain prevent LPS-induced inflammation (176). Interestingly, blocking inflammatory signaling increases APOE expression in microglia (177), suggesting a negative feedback loop between APOE levels and inflammation.

The mechanistic role of APOE expression in hypothalamic microglia has not been explored in models of diet-induced obesity, but data from studies in neurodegenerative disease lend clues towards the potential function.

### Triggering Receptor Expressed on Myeloid Cells 2 (TREM2)

The Triggering Receptor Expressed on Myeloid Cells 2 (TREM2) is a type 1 transmembrane receptor protein expressed on myeloid cells. This receptor binds a wide array of ligands including extracellular lipids and lipoproteins, and loss of function variants in TREM2 are also associated with increased risk of AD. TREM2 modulates inflammatory signaling in myeloid cells, and in the brain is primarily expressed by microglia. TREM2 is crucial for induction of the transcriptomic and functional program of DAMs, by activation of phagocytosis and lipid metabolism-related pathways. TREM2-deficient microglia have strong metabolic defects, characterized by impaired lipid metabolism, accumulation of cholesterol esters, aberrant autophagy, altered mTOR signaling, and reduced ATP production (8, 178). Also, TREM2-deficient microglia have reduced mitochondrial mass and increased phosphorylation of AMPK (178), a key regulator of energy metabolism that is activated in response to low glucose and inhibits a shift in the cellular metabolism from oxidative phosphorylation to glycolysis (179). A recent study revealed that TREM2 activation by APOE, drives a neurodegenerative phenotype in microglia, characterized by suppression of transcription factors regulating homeostatic microglia (171). Thus, targeting of the TREM2-APOE pathway may represent a novel therapeutic approach to restore homeostatic microglia in neurological disease.

TREM2 signaling in peripheral macrophages has recently been linked to metabolic disease. TREM2 KO mice exhibit increased obesity, insulin resistance and altered adipose tissue remodeling in response to HFD feeding (180). TREM2 is required for induction of monocyte-derived LAMs, in which LPL and APOE are induced by a TREM2-dependent mechanism as a consequence of HFD-induced obesity in mice (110). Similar transcriptional signatures were also identified in aortic macrophages during atherosclerosis (181) and fatty livers of mice fed a HFD (110). TREM2 activation *via* DAP12 antagonizes TLR signaling and inflammatory cytokine production in cultured macrophages and, conversely, TREM2 expression is abrogated by pro-inflammatory signaling (182, 183). However, the role of TREM2 in lipid-induced microglial activation in the MBH has not been investigated.

## Concluding Remarks and Future Perspectives

Both microglia and lipid metabolism are now known to play keys role in the onset and progression of the pathology of a wide variety of neurological diseases. The traditional view of the brain as an immune privileged organ has undergone a paradigm shift. In recent years, it has become increasingly clear that immune cells actively contribute to homeostatic processes in the CNS. Furthermore, dysfunctional microglial subsets characterized by excessive droplet- and membrane-associated lipid accumulation and attenuated lipid efflux have recently been the subject of considerable investigation (28, 184). Based on exciting data from other fields, it is increasingly becoming likely that a better understanding of how lipid mediators regulate the interaction between the immune and nervous systems may help uncover novel therapeutic targets to prevent and treat metabolic diseases as well. Indeed, many of the advances in determining of the role of lipid and lipoprotein metabolism that have occurred in the context of neurodegenerative disease (12) have the capacity to provide direct insight into the mechanisms by which microglia are activated in the MBH by nutritional signals.

The CNS hosts a heterogeneous population of myeloid cells, including parenchymal homeostatic microglia, and perivascular and meningeal border-associated macrophages. These myeloid cells share the expression of numerous markers, and a major obstacle has been the lack of tools to discriminate between specific microglial as well as other brain myeloid populations. However, new approaches for single-cell profiling have revealed a remarkable functional complexity in the CNS myeloid compartment in both homeostatic and disease contexts. Microglia are highly dependent on environmental signals to maintain their polarization. Given that such signals may vary across brain regions, it is notable that immune profiling of human brain microglia by single-cell proteomics revealed remarkable regional heterogeneity (185). Myeloid cells strategically located in close proximity to fenestrated blood vessels in the MBH may be able to sense metabolic factors including circulating lipids. To this end, we showed that HFD feeding induces the accumulation of a unique mix of myeloid cells in the MBH (15). This immunological response also includes the accumulation of perivascular macrophages involved in alterations systemic glucose metabolism (186). However, methods using marker-based analyses have technical limitations, and unbiased approaches are needed to resolve the heterogeneity and complexity of myeloid cell types within different CNS regions. Understanding the contribution of individual diet-responsive myeloid cell types will be critical for the development of novel therapeutics for obesity and T2D.

In summary, the emergence of a new field focused on microglial function, heterogeneity, and cell-cell crosstalk is providing us with an unprecedented understanding of how dietary lipids modulate microglial functions and their engagement with other cell types within the brain, including the MBH. This information has tremendous potential to help us identify new therapeutic targets to prevent overnutrition-induced hypothalamic dysfunction and metabolic disease.

## Author Contributions

All authors listed have made a substantial, direct, and intellectual contribution to the work and approved it for publication.

## Funding

This work was funded by the NIDDK (Diabetes, Endocrinology & Metabolism Training Grant T32DK007418 to AF, R01DK103175 to SK, K01DK113064, and R03DK125627 to MV).

## Conflict of Interest

The authors declare that the research was conducted in the absence of any commercial or financial relationships that could be construed as a potential conflict of interest.
